# microRNA biogenesis and senescence

**DOI:** 10.18632/aging.100607

**Published:** 2013-10-17

**Authors:** Daniel Gómez-Cabello, Isabel Adrados, Ignacio Palmero

**Affiliations:** ^1^ Instituto de Investigaciones Biomédicas “Alberto Sols” CSIC-UAM. Arturo Duperier, 4, E-28029 Madrid, Spain

Cellular senescence acts as a physiological barrier against uncontrolled proliferation of cells with potentially harmful alterations, contributing to tumor suppression and tissue homeostasis in live organisms [1]. Senescence is a complex phenotype that involves major changes in cellular function and structure. This phenotype is characterized by a specific gene expression program, which is controlled by key transcriptional regulators as well as by global and gene-specific epigenetic changes. In addition, the post-transcriptional control of senescence mediated by non-coding RNAs has recently attracted much interest [1]. In this context, our recent results [2] provide new evidence that supports the notion that microRNA-mediated control of gene expression plays a critical regulatory role in cellular senescence.

MicroRNAs are small noncoding RNAs that negatively regulate gene expression primarily through the degradation or translation inhibition of mRNAs with complementary sequences. To examine the impact of global disruption of miRNA activity in primary cells we focused on DGCR8 (also known as Pasha), an essential factor required for early steps of miRNA synthesis. Together with the ribonuclease DROSHA, DGCR8 forms the so-called Microprocessor nuclear complex, which is responsible of the processing of primary miRNA transcripts to generate the pre-miRNA intermediates that are further processed by Dicer to yield mature miRNAs [3]. We found that the disruption of miRNA biogenesis by silencing of DGCR8 behaved as a potent pro-senescence stimulus in human and mouse primary fibroblasts, leading to a clear reduction in proliferation, and the acquisition of classical senescence markers including flattened morphology, SA-BetaGal activity or formation of SAHF. Silencing of other key miRNA regulators, such as DROSHA or DICER had similar effects, supporting the notion that this phenotype was associated to global disruption of miRNA activity.

Our data ruled out that the induction of senescence observed could be due to indirect activation of oncogenes, DNA damage, or global changes in chromatin. Instead, we hypothesized that the silencing of the miRNA regulator DGCR8 could have subverted the physiological regulation of senescence by miRNAs. In our search for specific candidate miRNAs, we noted a significant overlap between miRNAs down-regulated during MEK-induced senescence and a set of miRNAs previously associated with cell-cycle defects in Dgcr8-defective ES cells [4]. This group of miRNAs included miR-20a, miR-106a and miR-93, a subset of miRNAs from the miR-17-92 family with identical seed sequences, and potentially common downstream targets. Interestingly, the miR-17-92 cluster and its paralogs have oncogenic features, since they are frequently overexpressed in tumors, and promote oncogenic transformation [5]. We found that this specific subset of miRNAs was down-regulated during senescence due to miRNA disruption, and the forced expression of miR-93 could overcome DGCR8-mediated senescence. Notably, it has been reported that several miRNAs of this group can also bypass senescence induced by the Ras oncogene in fibroblasts or epithelial cells [6, 7], highlighting the importance of these miRNAs as critical negative regulators of senescence.

Searching for downstream effectors of this response, we observed that the cdk inhibitor p21CIP1 was consistently increased during senescence due to DGCR8 knockdown. p21 is a direct transcriptional target of p53 and the role of the p53/p21 pathway in senescence is well known. In addition, p21CIP1 expression is also repressed by binding of specific miRNAs to its 3'UTR region. Of note, our subset of senescence-suppressive miRNAs were able to target the p21CIP1 mRNA for degradation, and the over-expression of miR-93 was capable of reverting, at least in part, the induction of p21 seen in DGCR8-deficient senescent cells. However, additional effectors must cooperate in the DGCR8-mediated response, since p21 was found to be dispensable, at least in murine fibroblasts. Indeed, we identified a number of additional cell-cycle genes differentially regulated during DGCR8-dependent senescence, which might play a role in the arrest. Interestingly, we also found that the anti-proliferative response to disrupted miRNA biogenesis is essentially retained in non-primary cells. In a limited survey of immortalized and tumor cell lines, we observed similar regulation of miRNAs, as well as p21CIP1, but intriguingly, a clear senescent phenotype was not induced in this case.

**Figure 1:**
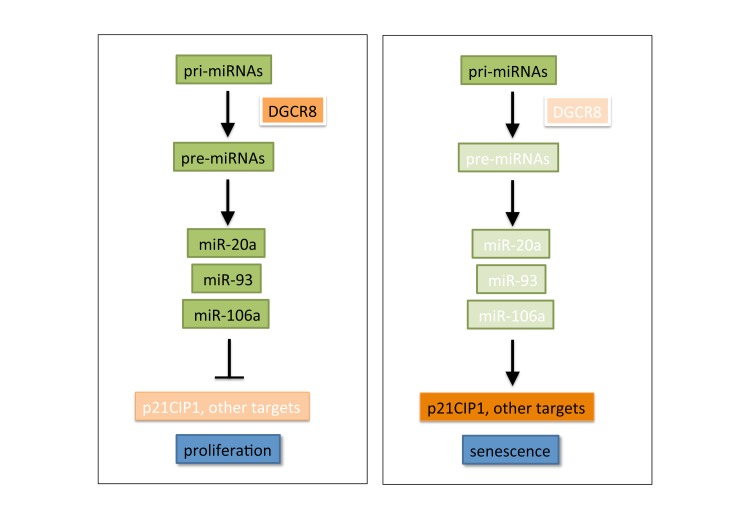
In proliferating primary cells, a proficient miRNA biogenesis machinery maintains the correct balance of miRNA expression, including high levels of senescence-suppressive miRNAs. Upon DGCR8-mediated disruption of miRNA biogenesis, senescence-suppresive miRNAs are down-regulated, leading to activation of a pro-senescent cascade. See text for details and discussion of alternative pathways.

Our results are consistent with the notion that global impairment of miRNA function is associated with reduced proliferation, in line with evidence from mouse models of defective miRNA regulators [4]. Although alternative pathways remain possible, our data suggest that senescence triggered by impaired miRNA biogenesis is primarily due to the reduction below a critical threshold of key senescence-suppressive miRNAs, which in turn trigger a downstream pro-senescent cascade. Our findings thus provide new evidence in support of the role of specific miRNAs as critical negative regulators of senescence. Several interesting questions remain open, including the involvement of additional downstream effectors, the crosstalk between pro and anti-senescence miRNAs, the differences between primary and tumor cell lines, or the potential role in this context of newly identified functions of DGCR8 beyond miRNA processing (see [8]). Given the emerging relevance of senescence in various physiological processes, it is increasingly important to understand the regulatory circuits that govern this response. miRNAs are clearly emerging as critical regulators of senescence, and further work lies ahead to fully unravel the complexity of miRNA-mediated regulation of cellular senescence.
